# Development of a PTFE membrane with photo-thermal activated carbon nanomaterials for improved solar-driven membrane distillation

**DOI:** 10.1039/d5ra06460k

**Published:** 2025-10-31

**Authors:** Wesam Abd El-Fattah, Ahlem Guesmi, Naoufel Ben Hamadi, Bassant Ebraheem, An Ding, Mohamed E. A. Ali

**Affiliations:** a Chemistry Department, College of Science, Imam Mohammad Ibn Saud Islamic University (IMSIU) P. O. Box 5701 Riyadh 11432 Saudi Arabia; b Egypt Desalination Research Center of Excellence (EDRC), Hydrogeochemistry Dept., Desert Research Center Cairo 11753 Egypt m7983ali@gmail.com; c School of Environment and Energy Engineering, Beijing University of Civil Engineering and Architecture Beijing 100044 China

## Abstract

Tackling global water scarcity requires effective desalination with renewable energy. This paper explores direct solar membrane distillation (MD). This technology uses photothermal nanoparticles. These nanoparticles capture sunlight and convert it into heat. This creates a thermal driving force at the membrane surface. This approach improves MD's energy efficiency. It also addresses temperature polarization. Polytetrafluoroethylene (PTFE) membranes with a PP backing layer were used. These were coated with membranes containing photothermally activated carbon (AC). The AC was integrated into polyvinyl alcohol (PVA) and glutaraldehyde (GA). GA acted as a cross-linker. The goal was to maintain water flow after coating. The performance of the PTFE/PVA–AC/GA membranes was tested. A synthetic saline solution was used. Adding hydrophilic PVA–AC improved the membrane's scaling resistance compared to PTFE. Increased PVA loading decreased water flow. The optimized PVA–AC–GA (0.25 wt% + 1 wt% + 1 wt%) membrane exhibited a stable vapor flux of 0.51 kg m^−2^ h^−1^ °C^−1^, which is comparable to the commercial PTFE membrane (0.58 kg m^−2^ h^−1^ °C^−1^), while providing enhanced photothermal activity and anti-wetting stability under simulated solar illumination. The membrane showed promising performance. They suit solar desalination off-grid for fluids prone to scaling.

## Abstract 2

Tackling global water scarcity requires effective desalination with renewable energy.

## Graphical abstract

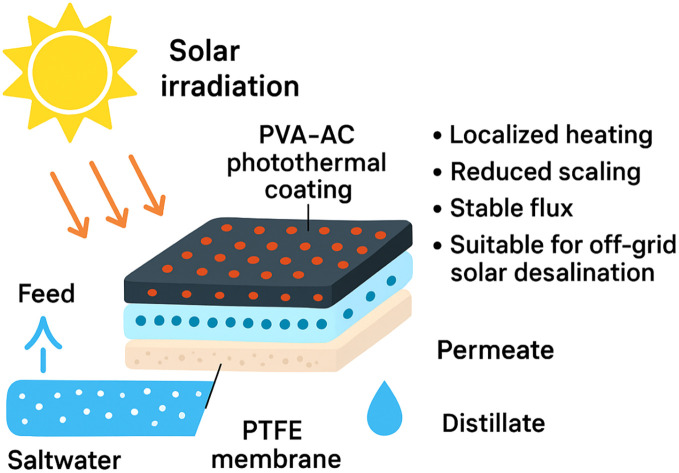

## 1 Introduction

Unprecedented global freshwater demands are a result of rapid economic development, population growth, and water pollution.^1–3^ Many countries use seawater and brackish water desalination. They do this to increase the freshwater supply. They also want to lessen water scarcity. Desalination has grown in recent decades. Seawater and brackish water make up 97.5% of the Earth's total water.^4–6^ More than 19 000 desalination plants globally were built by 2017, capable of producing approximately 100 million m^3^ per day.^7^ Water desalination uses different technologies. Some don't involve phase changes. Examples include electrodialysis (ED) and reverse osmosis (RO).^8–11^ Others use phase change processes. These include membrane distillation (MD) and thermal distillation (boiling). A thermally driven membrane technology with benefits is membrane distillation.^12,13^ It produces clean water using vapor pressure differences across a porous hydrophobic membrane.^14,15^ Direct contact membrane distillation (DCMD) is a common MD configuration. In DCMD, water evaporates on the hot feedwater side. It then diffuses across the membrane. Finally, it condenses on the cold distillate side.^16^ MD can operate at lower temperatures than boiling. It can also operate at lower pressures than RO.^17^ This leads to less electricity use. It also results in fewer fouling or corrosion problems.^14^ MD's compact footprint, modularity, and size stem from its basic equipment and pretreatment.^14,17,18^

The implementation of renewable energy sources like power plant waste heat^14,19^ and solar thermal collection systems^20,21^ to heat saline feed water gives an even greater incentive to use MD for sustainable water desalination. Standard MD, however, experiences temperature polarization, which reduces the surface temperature where the membrane meets the feed water, compared to the bulk water.^16,22^ Consequently, when the membrane has a smaller temperature difference, the MD performance is reduced.^23^ Lately, temperature polarization concerns^23,24^ are reduced *via* light-driven localized heating at membrane surfaces with photothermal materials (*e.g.*, carbon black, nitrocellulose, and Ag nanoparticles). By integrating photothermal materials, incident light, notably renewable solar irradiation, generates localized heating efficiently, which helps to elevate and sustain the membrane surface temperature at the interface of the membrane and feed water. The MD system with photothermal membranes notably lowers electricity use and has benefits like less fouling and easy system combination.^14^ Nevertheless, current photothermal materials have limitations, obstructing advancement and commercial use. Ag nanoparticles, for instance, tend to delaminate or leak from membranes into water.^23^ Dissolving photothermal material hinders its application in certain MD configurations, such as vacuum membrane distillation, and potentially degrades membrane photothermal performance over time. Solar energy is especially valuable for small off-grid setups and can be combined with MD by either using solar ponds or thermal collectors to heat the majority of the water,^24,25^ or by directly heating the membrane interface using a photothermal coating on the MD membrane.^26,27^ This second approach improved energy efficiency in MD. It uses the reverse temperature polarization profile from the self-heating membrane surface.^26^ An effective photothermal MD coating needs light-absorbing materials. These materials include plasmonic nanoparticles (NPs) and carbon-based materials. Activated carbon (AC) is an example. They must be in a coating layer. This layer permits the passage of water. Thermal energy is also captured from the photothermal agent. This indicates an equilibrium between coating thickness, boost light absorption, and water vapor flux, influenced by the coating's mass transfer resistance.^27–29^ Many studies have investigated the concurrent use of activated carbon (AC) with ultrafiltration, RO, and DCMD.^30–33^

The studies demonstrate that AC can effectively adsorb many different pollutants. Yet, they need more layers, making them less effective in flux and price. Alternatively, the membrane structure can directly incorporate AC for use as an *in situ* adsorbent. This approach offers several benefits, such as better porosity,^33,34^ effective *in situ* contaminant adsorption,^35^ and value-added product creation instead of incurring further expenses. Most studies employing AC incorporation have focused on polymeric membranes, such as polyvinylidene fluoride (PVDF) or polysulfone (PSf),^36–39^ which can be surface-modified or blended to achieve hydrophilic characteristics when used as composite or dual-layer membranes.^40–42^

However, despite the remarkable progress in photothermal membrane distillation (MD), most studies have mainly focused on either photothermal enhancement or anti-scaling improvement individually. Limited research has explored a combined approach that simultaneously integrates both properties within a single, structurally stable coating. Moreover, conventional dip-coating or blending methods often lead to thick or uneven layers that compromise vapor flux and long-term performance.

In this work, a novel dual-functional membrane is developed by applying a thin, uniform PVA–AC (polyvinyl alcohol–activated carbon) layer *via* spray-coating onto a hydrophobic PTFE substrate. This design provides a hydrophilic surface that enhances anti-scaling resistance while the embedded activated carbon functions as a photothermal agent for localized solar heat generation. To the best of our knowledge, this is one of the first systematic studies to investigate the combined effects of PVA and AC concentrations on both photothermal performance and water flux stability in solar-driven MD systems.^28^

## 2 Experimental

### 2.1. Materials and reagents

A hydrophobic polytetrafluoroethylene (PTFE) membrane with a polypropylene (PP) support and a nominal pore size of 0.45 μm was obtained from Jian City Qing Feng Filter Equipment Material Co., Ltd (China) and used as a substrate for the PVA/activated carbon (AC) composite layer. ACROS Organics supplied the poly(vinyl alcohol) (PVA) used, which had a molecular weight of 85 000–124 000 and a 95.5–96.5% hydrolysis degree. Activated carbon (AC) was sourced from India. Glutaraldehyde (GA) solution (50% in water) was supplied by ADWIC (Egypt) and used as a crosslinking agent. Additional chemicals, including acetone, ethyl alcohol, and hydrochloric acid (36–37%), were procured from PIOCHEM (Egypt). Synthetic feed solutions for membrane distillation (MD) testing were prepared using deionized water (LUNDA type PD 8 R, Germany) and analytical-grade salts: NaCl, CaCl_2_, KCl, Na_2_SO_4_, and MgSO_4_·7H_2_O. No purification was performed before the chemicals were used.

### 2.2. Coating the PTFE membranes

Spray-coating was used to deposit thin films of PVA and PVA–AC composites on PTFE membrane substrates. To enhance film adhesion, PTFE membranes underwent pretreatment with 5 mL of ethanol to clean and impart partial hydrophilicity to their surface before deposition. Different concentration PVA solutions (0.25, 0.5, 0.75, and 1 wt%) were created by dissolving PVA in a 2 : 3 water/ethanol mix at 200 °C, under constant stirring for 2 hours. For the preparation of PVA–AC composite solutions, activated carbon (AC) was added to the PVA solution at the corresponding concentrations (0.25, 0.5, 0.75, and 1 wt%), followed by sonication for 1 hour to ensure homogeneous dispersion.

The PTFE membrane surface was sprayed with the resulting solutions using a Master Airbrush spray nozzle (Model G22) powered by a 1/5 HP air compressor at a steady 40 psi. The thickness of the coated layer was controlled by adjusting the volume of the sprayed solution. Following deposition, the membranes were air-dried at an ambient temperature for 10 minutes. Crosslinking was then carried out by spraying a solution containing 2% glutaraldehyde (GA) in a 2 : 3 acetone/water mixture, using the same spray system, catalyzed by 0.12 mol per L HCl. We allowed the membranes to dry at room temperature overnight.

The crosslinked films received thorough ethanol and distilled water rinsing to eliminate residual GA and HCl. Membranes were held at room temperature until future use.

**Scheme 1:**
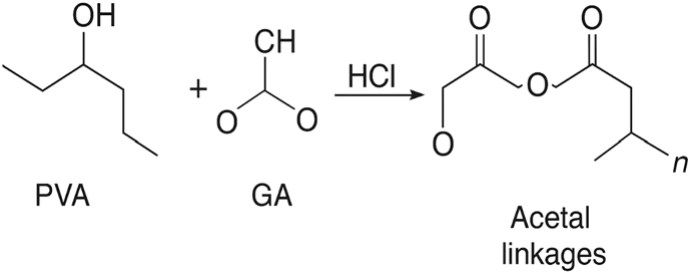
Crosslinking mechanism between PVA and GA under acidic conditions forming acetal linkages that stabilize the PVA–GA coating and embed AC particles.

During the crosslinking step, glutaraldehyde (GA) reacts with the hydroxyl groups (–OH) of PVA under acidic conditions to form acetal linkages, as illustrated in the Scheme 1. This reaction produces a stable three-dimensional PVA–GA network that firmly immobilizes the activated carbon (AC) particles within the coating. The reaction can be represented as:PVA–OH + OHC–(CH_2_)_3_–CHO → PVA–O–CH–(CH_2_)_3_–CH–O–PVA + H_2_O

Although PTFE is chemically inert, the ethanol-based PVA–AC suspension enhances interfacial wettability during spray coating by reducing surface tension, allowing the coating solution to spread uniformly. Upon solvent evaporation, the PVA film becomes physically anchored to the PTFE substrate through van der Waals forces and surface interlocking, ensuring stable adhesion of the composite layer.

### 2.3. Bench-scale direct contact MD experiments

**Fig. 1:**
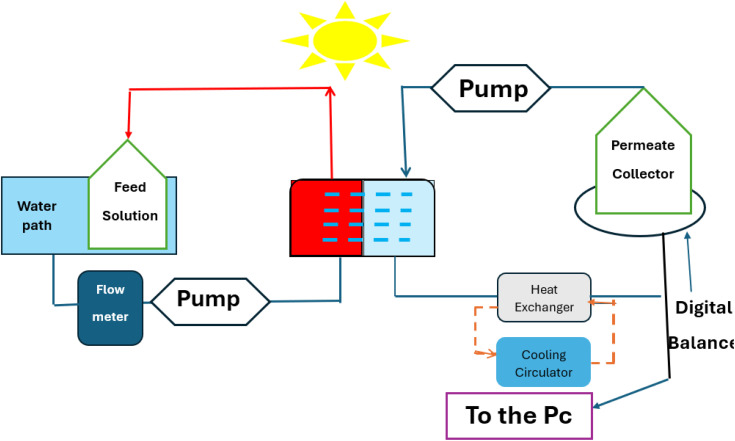
Schematic of the direct contact membrane distillation (DCMD) apparatus used for photothermal testing under simulated solar illumination.

The photothermal membrane's effectiveness in direct contact membrane distillation mode was tested using a bench-scale nanophotonically Enhanced Solar Membrane Distillation system, illustrated in Fig. 1. A flat-sheet configuration was used for the membrane module, with an effective membrane area of 0.0036 m^2^. The 2 mm channel height was consistently applied in the design of the feed and distillate flow channels. A quartz window was used to allow direct sunlight to hit the membrane. A photograph of the original DCMD test cell and the quartz window used for illumination is provided in the SI (Fig. S1).

To mechanically support the membrane and distribute flow evenly, polypropylene mesh spacers were used on both sides. The feed and permeate streams were circulated at a constant flow rate of 150 mL min^−1^ using a peristaltic pump, corresponding to an estimated linear flow velocity of approximately 0.2 m s^−1^, based on the internal dimensions of the flow channels. Inlet and outlet temperatures of both streams were continuously monitored using calibrated thermocouples to track thermal gradients across the membrane module.

A synthetic saline solution (1 L) was used as the feed, with its composition detailed in Table S1 (see SI). It was prepared to simulate the average ionic composition of natural seawater (total salinity ≈35 g L^−1^). The solution contained NaCl, Na_2_SO_4_, NaHSO_4_, MgCl_2_·6H_2_O, KCl, and CaCl_2_ in specific proportions, as listed in Table S1. This formulation was adopted from previously reported seawater compositions commonly used in membrane distillation studies to ensure realistic salinity conditions.^39^

The solution was progressively concentrated throughout the experiment. Initially containing 400 mL of deionized water, the permeate reservoir was set on a digital balance connected to a computer for constant data logging. The permeate mass was recorded at regular intervals to monitor system output. The following equation was used to calculate the direct contact membrane distillation (DCMD) flux, *J* (kg m^−2^ h^−1^).^40^1
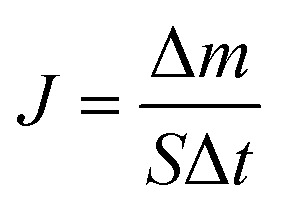
where Δ*m* signifies the permeate mass increase, measured in kg, over a specific period; The salt rejection, *R*_s_ (%), was determined according to the method described in ref. 41, by calculating the salt concentrations in the hot feed (*C*_f_) and the permeate (*C*_p_) using the following equation:2
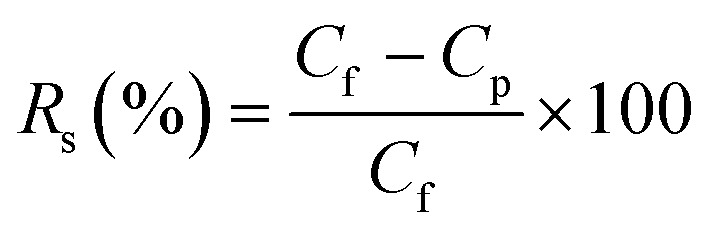


Feed water was supplied at 200 mL min^−1^ and 70 °C for the MD experiments, with the distillate side at 10 °C (Δ*T* = 60 °C). DI water with 1% HCl was used to clean the system between experiments; then, only DI water was used for rinsing.

All DCMD experiments were carried out using a flat-sheet test cell with an effective membrane area of 36 cm^2^. Both feed and distillate streams were circulated at 150 mL min^−1^. The feed temperature was maintained at 70 °C and the distillate at 10 °C (Δ*T* = 60 °C). For solar-driven tests, the feed and distillate were kept at 40 °C and 10 °C (Δ*T* = 30 °C), respectively, under a low-intensity illumination of 36 W m^−2^ (≈0.036 sun, 3.6% of AM 1.5G). Each experiment lasted 1200 minutes, and permeate mass was recorded every 1 minute.

The schematic illustrates the DCMD test cell configuration, showing that the bulb light source (100 and 200 W) was positioned above the feed side of the membrane module. The incident light irradiated the feed-side surface of the PVA–AC-coated membrane through the transparent quartz window, allowing photothermal heating of the membrane surface in direct contact with the saline feed stream. The permeate side remained thermally isolated to ensure that the observed enhancement originated from the photothermal activity of the coated layer.

### 2.4. Photothermal experiments

In the solar membrane distillation (MD) experiments, the feed temperature was held at 40 °C, establishing a baseline temperature of approximately 30 °C within the system. A cold-water bath containing glass coils maintained the distillate stream at 10 °C by circulation. In the solar MD experiments, the feed and distillate were maintained at 40 °C and 10 °C (Δ*T* = 30 °C), respectively. Illumination of the membrane was achieved using 100 W and 200 W lightbulbs positioned 13.5 cm above the transparent membrane cell. This setup produced incident light intensities of 100 and 200 mW cm^−2^ at the membrane surface, as transmitted through a clear acrylic cover. For precise irradiation quantification on the membrane, a Thorlabs PM100D power meter (Lübeck, Germany) was used to measure light intensity.

## 3 Results and discussions

### 3.1. Structural of activated carbon properties

Scanning electron microscopy (FEI Quanta FEG 250) with energy-dispersive X-ray spectroscopy (EDX) was used to analyze the elemental composition and microstructural features of the activated carbon (AC) samples. The elemental percentages obtained from the EDX analysis are summarized in Table S2 of the SI.

**Fig. 2:**
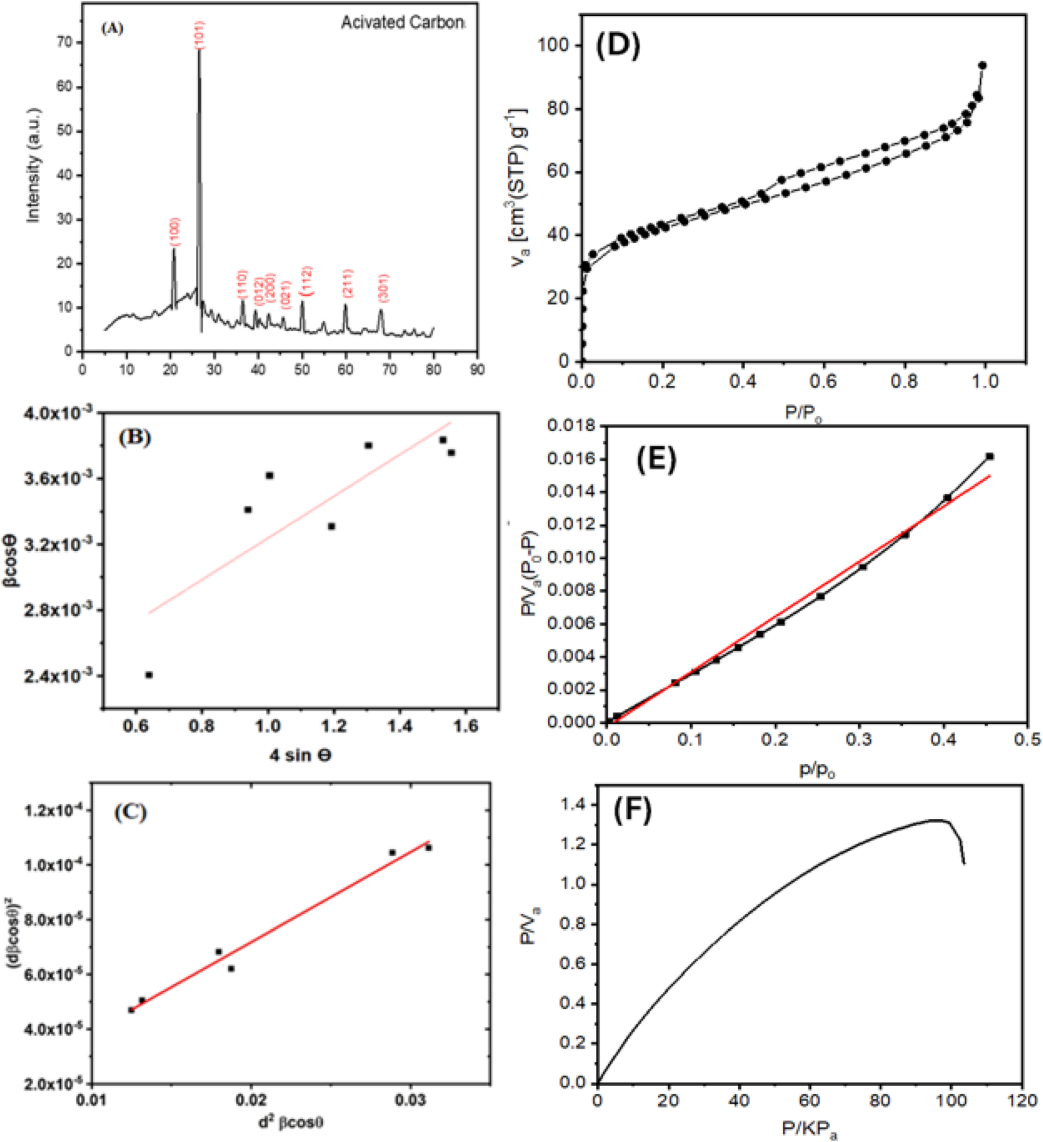
(A) X-ray diffraction pattern of powder activated carbon, (B) plot of (*β* cos *θ*) *versus* and (4 sin *θ*), (C) plot of (*dβ* cos *θ*)^2^*versus* (*d*^2^*β* cos *θ*), (D) the nitrogen adsorption isotherm of the AC, (E) BET plot of the AC, (F) the adsorption behavior of the AC using the Langmuir isotherm.

X-ray powder diffraction (XRD) was used to explore the AC powder's crystalline structure, and the resulting diffraction pattern is in Fig. 2A. Multiple peaks of varying intensities were observed, indicating that the AC sample is polycrystalline. The diffraction data confirm that the material adopts a hexagonal crystal structure, consistent with space group *P*3_1_21 (No. 152). The refined lattice parameters were found to be: *a* = *b* = 4.92 Å, *c* = 5.41 Å, with a unit cell volume of 113.59 Å^3^ and a theoretical density of 2.635 g cm^−3^.

Scherrer's equation^42^ was employed to calculate the average crystallite size (*D*) of the AC, utilizing the FWHM of the (101) diffraction peak, which was the most intense in the XRD pattern, as follow.3
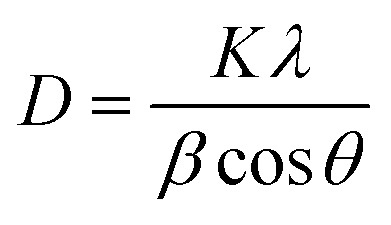
where *K* is the Scherrer constant (0.9), *λ* is the X-ray wavelength (Cu Kα radiation, 1.5406 Å), *β* is the full width at half maximum (FWHM) of the diffraction peak (in radians), and *θ* is the Bragg diffraction angle. The measured *D* of AC value was 18.65 nm for the (101) diffraction peak. Moreover, the crystallite's average size *D*, and strain *ε* will be calculated using the Williamson–Hall (W–H) method. (W–H) analysis uses equation^43^ and a simplified integral breadth form to find *D* and *ε* by considering diffraction peak width dependence on 2*θ*°.4
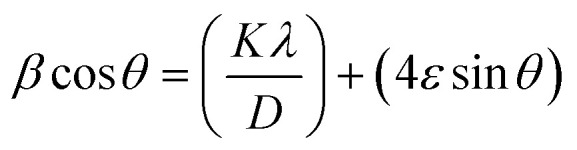


Fig. 2B presents the relation between (*β* cos *θ*) and (4 sin *θ*). *ε* and *D* for AC form were derived from the slope and intercept of the linear fit to the experimental data and are in Table 1. The obtained structural parameters of AC reflect its crystalline perfection, which supports its efficient photothermal behavior when incorporated into the membrane coating used in the DCMD system.

**Table 1:** Crystallite size and lattice strain of activated carbon (AC) nanoparticles calculated *via* Scherrer, Williamson–Hall (W–H), and size–strain plot (SSP) methods

Williamson–Hall method (W–H)	*D* (nm)	36.6
	*ε* × 10^−3^	1.37
Size–strain plot method (SSP)	*D* (nm)	22.9
	*ε* × 10^−3^	2.9
Scherrer method	*D* (nm)	29.75

Isotropic line broadening was shown by the W–H plots. A typical size–strain plot (SSP) also helps create a more exact estimation of size–strain parameters. In this system, the ‘crystallite size’ profile is modeled as a Lorentzian, while the ‘strain profile’ is modeled as a Gaussian, as given by.^44^5
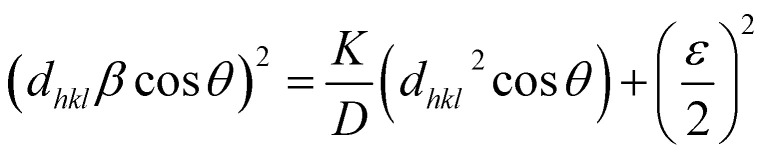


The plot of (*dβ* cos *θ*)^2^ compared to (*d*^2^*β* cos *θ*) is shown in Fig. 2C. After a linear fit to the data, AC crystallite size and strain values were determined and presented in Table 1. Comparable vibrations in average crystallite size values can be observed across these various methods. Hence, AC nanoparticle average crystallite size is affected by the strain in several forms. The strain values are 1.37 × 10^−3^ and 2.9 × 10^−3^ using W–H and SSP, respectively. Additionally, the dislocation density, *d*, is determined by calculating the length of dislocation lines per unit volume using the following equation.6
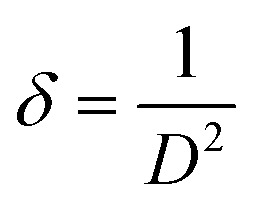


The strain (*ε*) calculated from XRD refers to the AC powder and is dimensionless (no unit). The relatively low strain values indicate low lattice distortion and high crystallinity, which enhance the photothermal stability and performance of the AC-based composite membranes.

The XRD analysis was carried out to determine the crystallite size and lattice strain of the activated carbon (AC) powder used as the photothermal additive. Eqn (4) and (5) correspond to the Williamson–Hall (W–H) and Size–Strain Plot (SSP) methods, respectively, which were employed to separate the effects of crystallite size and microstrain on the peak broadening. These analyses help in correlating the structural perfection and defect levels of AC with its optical and photothermal properties.

The specific surface area was determined using the linear part of the BET plot, while adsorption isotherms were measured between 0.05 and 0.3 *P*/*P*_0_. The nitrogen adsorption isotherm of the AC, as shown in Fig. 2D, exhibits a typical type IV isotherm according to the IUPAC classification. The isotherm displays a nearly flat adsorption region at low relative pressures (*P*/*P*_0_ < 0.8), followed by a sharp increase in the adsorbed volume at high relative pressures (*P*/*P*_0_ > 0.9). This behavior is characteristic of mesoporous materials, indicating the presence of well-developed mesopores within the sample. The steep uptake at high *P*/*P*_0_ values is attributed to capillary condensation occurring in the mesopores. The specific surface area was calculated *via* the Brunauer–Emmett–Teller (BET) method using the linear region within the *P*/*P*_0_ range. The high specific surface area and mesoporous structure confirmed by these results make the sample suitable for catalysis and adsorption applications.

The sample's specific surface area was determined using the BET method. The appropriateness of the BET model is demonstrated by the BET plot Fig. 2E, which shows a linear relationship in the relative pressure. The monolayer capacity and the BET constant were computed using the slope and intercept derived from the linear region. The BET surface area of 149.1 m^2^ g^−1^ confirmed the synthesized material's high surface area and porosity. The sample's specific surface area was determined using the BET method, Table 2.

**Table 2:** Brunauer–Emmett–Teller (BET) analysis results for activated carbon (AC): specific surface area, monolayer volume (*V*_m_), total pore volume, and average pore diameter

Parameters	Value
*V*_m_	34.258
BET	149.11
Average pore diameter	3.7973
Total pore volume	0.1416

The Langmuir adsorption isotherm is a widely used model to describe the adsorption of gases onto solid surfaces, assuming monolayer coverage and a finite number of identical adsorption sites. The Langmuir model is mathematically expressed as:7
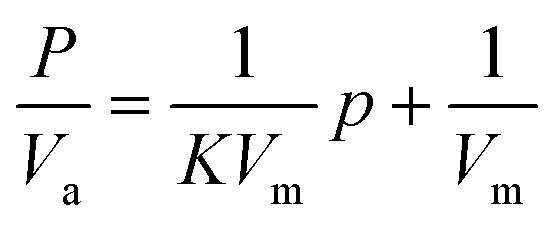
where: *p* = equilibrium pressure of the adsorbate (kPa). *V*_a_ = volume of gas adsorbed at pressure *p*. *V*_m_ = monolayer capacity (maximum adsorption). *K* = Langmuir adsorption constant (related to adsorption energy).

The adsorption behavior of the sample was analyzed using the Langmuir isotherm. The Langmuir plot Fig. 2F displays a linear relationship between *p*/*V*_a_ and *p* at low pressures, confirming the applicability of the Langmuir model for monolayer adsorption. From the slope and intercept of the linear region, the monolayer adsorption capacity *V*_m_ and Langmuir constant *K* were determined. Deviations from linearity at higher pressures suggest the presence of multilayer adsorption or surface heterogeneity, which are beyond the scope of the Langmuir model.

The Langmuir isotherm model was used to assess the adsorption capacity and surface homogeneity of the activated carbon (AC) powder. These parameters reflect the strength and distribution of the active sites, which influence the membrane–water interfacial behavior and consequently the DCMD performance of the PVA–AC coated membranes.

**Fig. 3:**
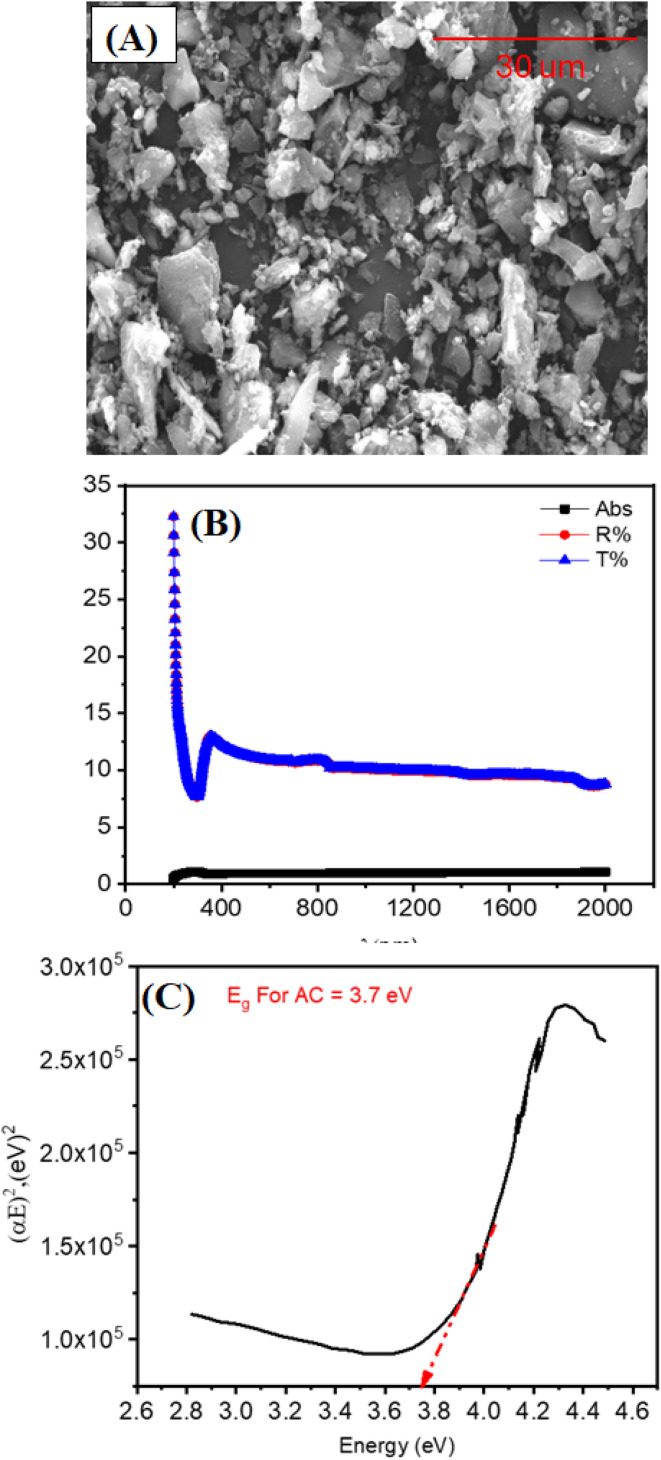
(A) SEM morphology of activated carbon (AC) nanoparticles; (B) optical transmittance (*T*), reflectance (*R*), and absorbance spectra of AC; (C) Tauc plot for direct band gap determination (*E*_g_ = 3.7 eV).

The surface morphology was investigated because it significantly impacts device performance. The acquired grains' size was calculated using SEM images, which also supported crystallinity. The AC structure's surface topography was investigated using SEM images and Image application results. Fig. 3A illustrates the SEM images' front view, which displays a high density and aggregation of grains across the surface, randomly distributed.

The investigation of optical properties offers critical insight into fundamental physical parameters, particularly the energy band gap. Fig. 3B shows the transmittance (*T*(*λ*)) and reflectance (*R*(*λ*)) spectral profiles for the activated carbon (AC) sample, which were measured at normal incidence in the 200–2000 nm range. The optical band gap (*E*_g_) of AC was determined from the absorption behavior using Tauc's method, which is applicable within the strong absorption region of semiconductors.^45^ According to Tauc's relation,^46^ the band gap can be extracted from the dependence of the absorption coefficient (*α*) on photon energy (*E*) *via* the following expression:

8 (*αE*)^2^ = *A*(*E* − *E*_g_)

As illustrated in Fig. 3C, a plot of (*αE*)^2^*versus* photon energy (*E*) was made for direct band gap estimation. The curve's linear part was extrapolated to the photon energy axis (*E*-axis), where it intersected, showing the direct optical band gap. The calculated value of *E*_g_ for the AC sample is summarized in Table 3.

**Table 3:** Experimental and theoretical optical band gap values (*E*_g_) of activated carbon (AC) from literature compared with the present work (3.7 eV)

Samples	Determination method	*E*_g_ (eV)	Ref.
AC	Experimental	3.7	Present work
AC	Experimental	3.15	46
AC	Experimental	3.10	46
AC	Experimental	2.80	46
AC	Experimental	2.82	46
AC	Theoretical	1.98	47
AC	Theoretical	2.52	47
AC	Theoretical	2.83	47
AC	Theoretical	2.78	47
AC	Experimental	3.15	48
AC	Experimental	3.68	48
AC	Experimental	2.83	48

An energy gap of 3.7 eV corresponds to a maximum absorption wavelength (*λ*_max_) calculated as:9
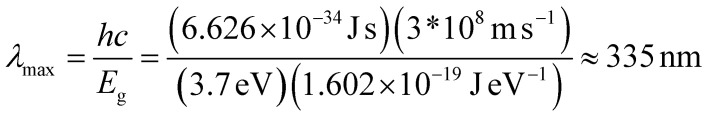
where *h* is Planck's constant (6.626 × 10^−34^ J s), *C* is the speed of light (3 × 10^8^ m s^−1^), and *λ*_max_ is the wavelength corresponding to the maximum absorbance in the UV-Vis spectrum. The ultraviolet (UV) region of the electromagnetic spectrum includes this wavelength. While the AC thin film from the present work will absorb UV light strongly, which might be advantageous for specific niche photothermal applications utilizing UV light sources or where selective UV absorption is desired.

### 3.2. Characterization of the photothermal membrane coating

A thin microporous PTFE layer sits atop a PP microfiber support in this membrane. This configuration is advantageous for membrane distillation (MD) due to the intrinsic hydrophobicity of PTFE, reflected in a high water contact angle (WCA) of 103.05 ± 2.4°, attributed to its surface functional groups.^49^ However, the inherent low adhesion of poly(vinyl alcohol) (PVA) to the PTFE surface poses challenges for conventional coating methods such as dip coating.

**Fig. 4:**
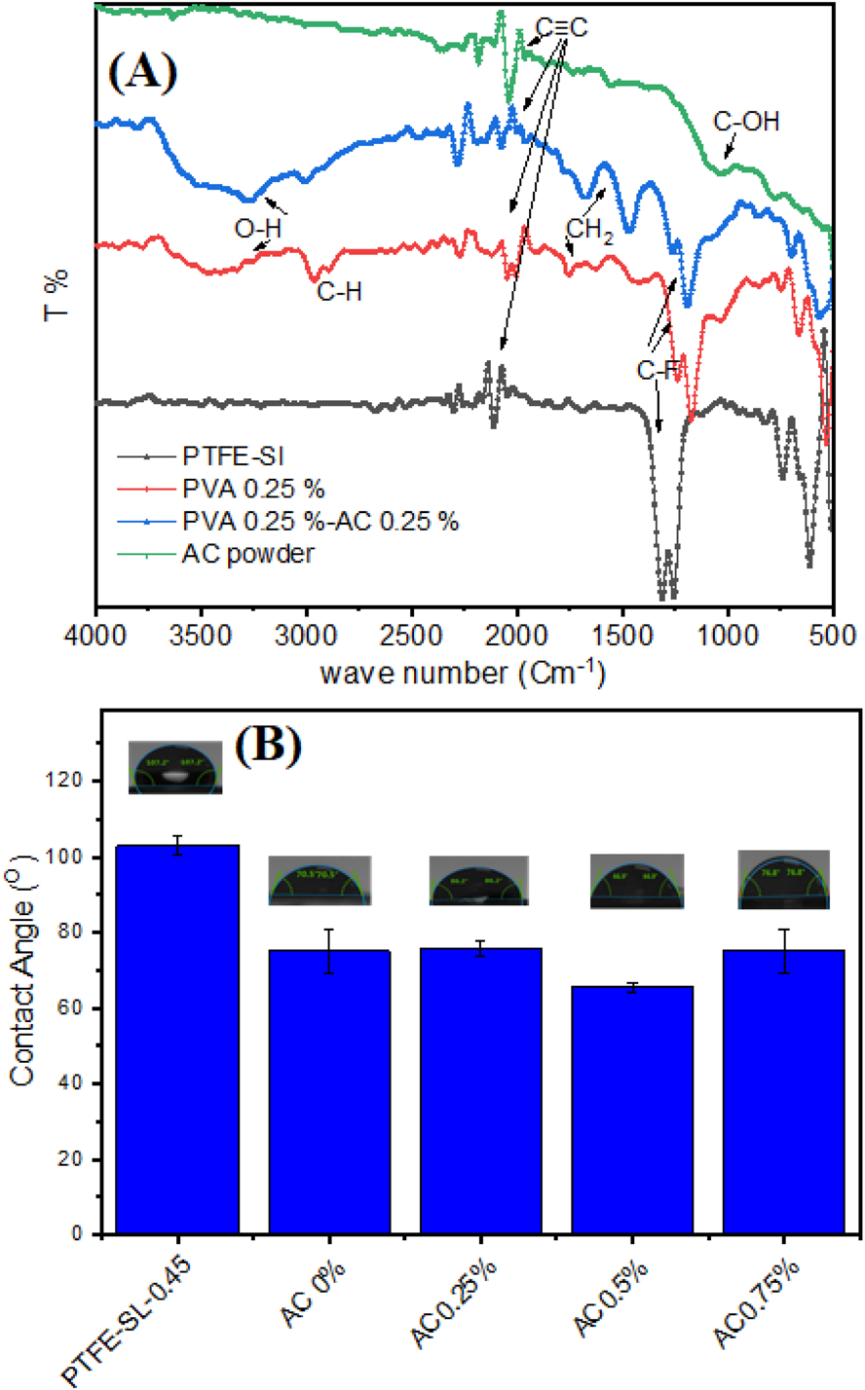
FTIR spectra of PTFE commercial membrane, PVA 0.25% wt, PVA 0.25% wt–AC 0.25% wt, PVA 0.25% wt–AC 0.5% wt, and PVA 0.25% wt–AC 0.75% wt (A), surface hydrophilicity, measured by WCA analysis for pristine PTFE and coated membrane with PVA 0.25% wt and different concentration of AC (B).

In order to combat this issue, a particular coating method for applying a photothermal PVA–AC (activated carbon) layer to the PTFE membrane was devised. The process began with a surface pre-treatment step, followed by the application of the PVA–AC layer. The use of a water : ethanol mixture as a dispersion medium for AC was found to enhance dispersion stability, which is consistent with findings for similar nanomaterials like graphene in aqueous-ethanol systems.^50^ A drop in the media's polarity is the principal reason for this improvement. Also, ethanol's reduced surface tension promotes partial wetting of the PTFE surface, which helps the coating layer adhere. These combined effects enabled the formation of a uniform and stable PVA–AC film on the PTFE membrane. Fourier-transform infrared (FT-IR) spectroscopy was used to investigate the chemical interactions and coating composition. FTIR spectroscopy was employed to probe the chemical structures and surface functional groups of the pristine PTFE membrane, PVA-coated membrane (0.25%), PVA–AC composite membrane (0.25–0.25%), and raw activated carbon (AC) powder. The resulting spectra, shown in Fig. 4A, span the wavenumber range from 4000 to 500 cm^−1^. The spectrum of the pristine PTFE membrane (black curve) displays prominent absorption bands in the region of 1200–1100 cm^−1^, which correspond to C–F stretching vibrations characteristic of the fluorinated polymer backbone. Notably, the absence of broad absorptions in the higher wavenumber region (above 3000 cm^−1^) confirms the non-polar, chemically inert nature of PTFE, as expected.

In contrast, the PVA-coated membrane (red curve) exhibits a broad, intense absorption band between 3300 and 3500 cm^−1^, indicative of O–H stretching vibrations from hydroxyl groups. This is a hallmark of hydrogen bonding within the PVA matrix. Additionally, a peak near 2900 cm^−1^ arises from the C–H stretching of aliphatic –CH_2_ groups. The presence of bands around 1420 cm^−1^ and 1090 cm^−1^ corresponds to CH_2_ bending and C–O stretching, respectively, confirming the successful deposition of PVA onto the PTFE surface. With the incorporation of activated carbon, the PVA–AC composite membrane (blue curve) retains the key spectral features of PVA, but with increased intensity in the O–H stretching region. This enhancement may reflect additional hydroxyl groups or physisorbed water introduced by the porous AC structure. Minor alterations in the fingerprint region (1700–1000 cm^−1^), for example, bands that are new or displaced, point to the possibility of PVA chains interacting with AC surface functionalities through van der Waals forces, hydrogen bonding, or π–π stacking.

The spectrum of pure AC powder (green curve) is dominated by a broad O–H stretching band centered near 3430 cm^−1^, which can be attributed to surface hydroxyl groups and/or adsorbed moisture. A distinct peak at approximately 1600 cm^−1^ is characteristic of C=C stretching vibrations in aromatic domains. Additional features in the 1200–1000 cm^−1^ range are likely associated with C–O and C–OH stretching vibrations from oxygen-containing surface groups commonly found on activated carbon. Taken together, these observations confirm the effective functionalization of the PTFE substrate with PVA and PVA–AC layers, while also preserving the chemical identities and interactive potential of the individual components. The presence of characteristic bonds across all samples supports the structural integrity of the fabricated composite membranes.

Water contact angle measurements Fig. 4B further revealed the wettability changes induced by the coating. While the pristine PTFE membrane exhibited strong hydrophobicity, coating with PVA reduced the WCA to 72°, indicating increased hydrophilicity due to the polar nature of PVA. Upon the incorporation of AC into the PVA matrix, the WCA increased again, a result of the hydrophobic characteristics of AC, which contain a high concentration of –CH_2_ and other non-polar functional groups. The WCA decreased from 110 ± 2° for pristine PTFE to 72 ± 3° for the PVA-coated membrane, and then increased to 92 ± 2° after the incorporation of AC. The rise in WCA reflects the combined effects of AC's hydrophobic domains and the enhanced surface roughness of the composite coating. The decrease in contact angle observed at 0.5 wt% AC is attributed to the uniform distribution of AC nanoparticles within the PVA layer, which enhances surface hydrophilicity and roughness. Beyond this concentration, aggregation of AC reduces the effective hydrophilic area, increasing the contact angle.

**Fig. 5:**
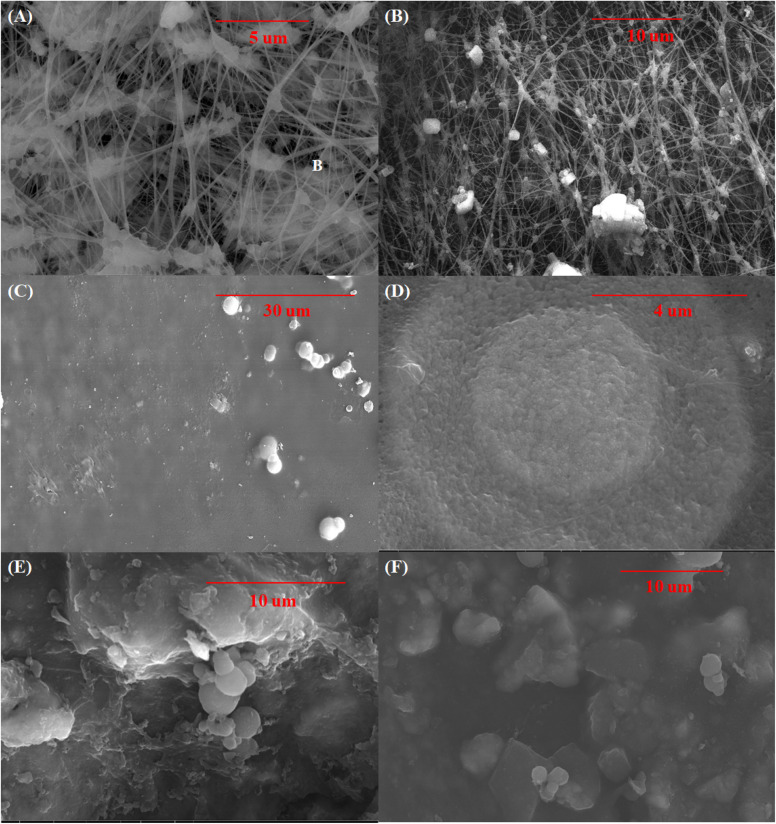
(A) SEM images of the pristine (PTFE) membrane, (B) Pristine after scaling up, (C) PVA 0.25% wt-coated membrane, (D) PVA 0.25% wt–AC 0.25% wt coated membrane, (E) PVA 0.25% wt–AC 0.5% wt coated membrane, (F) and PVA 0.25% wt–AC 0.75% wt coated membrane.

SEM was used to study the coating morphology on the surface Fig. 5. The typical fiber-nodule pore structure characterizes the commercial PTFE membrane Fig. 5A, also contributing to the surface's hydrophobicity. Also, the surface after scaling was Studied which observed the salt retention and deposition on the surface as shown in Fig. 5B. PVA addition hides the reticular structure, forming a uniform, defect-free layer on the PTFE membrane Fig. 5C. The PVA layer deforms, pores emerge in the coating, and AC particles are visible as spheres throughout the PVA–AC coating, all due to the addition of AC particles acting as a photothermal agent (Fig. 5D–F). Table S3 elucidates the ratio of ions deposition on the membrane surface before and after MD performance, which observed the high quality of membrane to reject salts. Although the SEM images show a uniform surface layer, the deposited PVA–AC film is ultrathin and maintains nanoscale voids that permit vapor diffusion while inhibiting liquid entry. This structure ensures that the DCMD process operates through vapor-phase transport rather than liquid penetration. Despite the seemingly smooth surface in SEM images, the ultrathin and hydrophilic PVA–AC layer enables vapor transmission through its polymeric network, maintaining the DCMD functionality while resisting pore wetting.^51–54^

### 3.3. MD performance

**Fig. 6:**
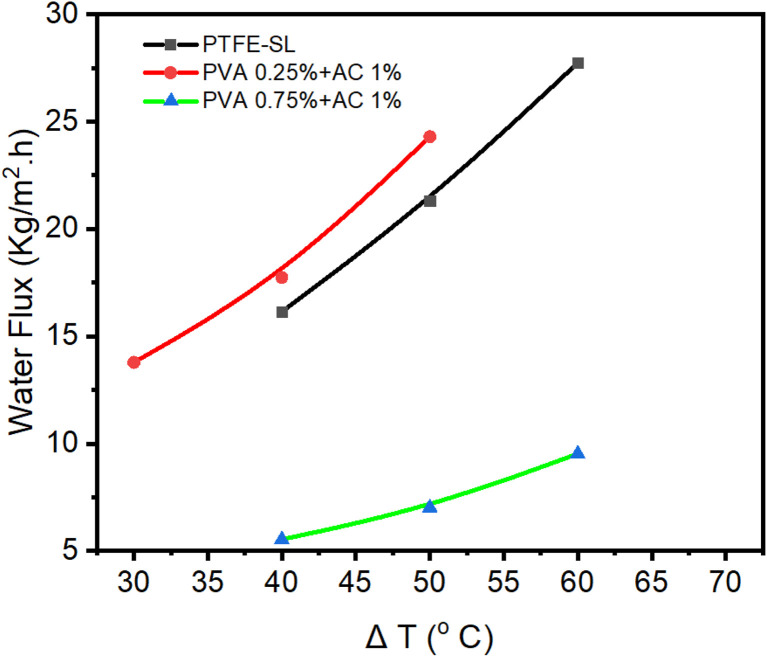
Permeability coefficient for PTFE membrane without any coating, for membrane coated with PVA 0.25 wt%, AC 1 wt% and GA 1 wt%, and for membrane coated with PVA 0.75 wt%, AC 1 wt% and GA 1 wt%.

The pure water permeability (PWP) of the membranes was assessed using distilled water at different feed temperatures. The permeability coefficient was obtained from the slope of the linear fit, as shown in Fig. 6 for three reasons: the uncoated PTFE membrane, the membrane coated with 0.25% wt PVA + 1% wt AC + 1% wt GA, and the membrane coated with 0.75% wt PVA + 1% wt AC + 1% wt GA. The corresponding values are summarized in Table 4. Fig. 6 illustrates the relationship between water flux and the temperature difference (Δ*T*) across the three membrane types: PTFE-SL, PVA 0.25% wt + AC 1% wt + GA 1% wt, and PVA 0.75% wt + AC 1% wt + GA 1% wt. In all cases, water flux increased with Δ*T*, reflecting the higher vapor pressure gradient between the feed and permeate sides. The slopes of the fitted lines, representing the permeability coefficients, were 0.58, 0.53, and 0.2 kg m^−2^ h^−1^ °C^−1^ for PTFE–SL, PVA 0.25 wt% + AC 1 wt% + GA 1 wt%, and PVA 0.75 wt% + AC 1 wt% + GA 1 wt% membranes, respectively. Although the permeability of the PVA 0.25 wt% + AC 1 wt% + GA 1 wt% membrane (0.51 kg m^−2^ h^−1^ °C^−1^) was slightly lower than that of the commercial PTFE–SL (0.58 kg m^−2^ h^−1^ °C^−1^), it still demonstrated comparable vapor transport performance while offering enhanced photothermal activity and anti-wetting stability due to the synergistic effect of AC incorporation and crosslinking with GA.

**Table 4:** Permeability coefficient for PTFE membrane without any coating and coated with different concentration

Membrane type	Permeability coefficient
PTFE-S-0.45	0.58
PVA 0.25 wt% + AC 1 wt% + GA 1 wt%	0.53
PVA 0.75 wt% + AC 1 wt% + GA 1 wt%	0.2

Conversely, the reduced permeability observed for the PVA 0.75 wt% + AC 1 wt% + GA 1 wt% membrane can be attributed to the higher polymer concentration, which led to a denser and less porous structure that restricted vapor diffusion. Although the pristine PTFE membrane exhibited a slightly higher permeability coefficient, the PVA–AC–GA composite membrane showed superior photothermal and anti-wetting performance, achieving more stable vapor flux under solar-assisted operation.

While the pristine PTFE membrane exhibited a marginally higher flux, the PVA–AC–GA composite membrane demonstrated enhanced stability and photothermal efficiency under illumination, confirming its functional advantage in solar-assisted DCMD applications.

**Fig. 7:**
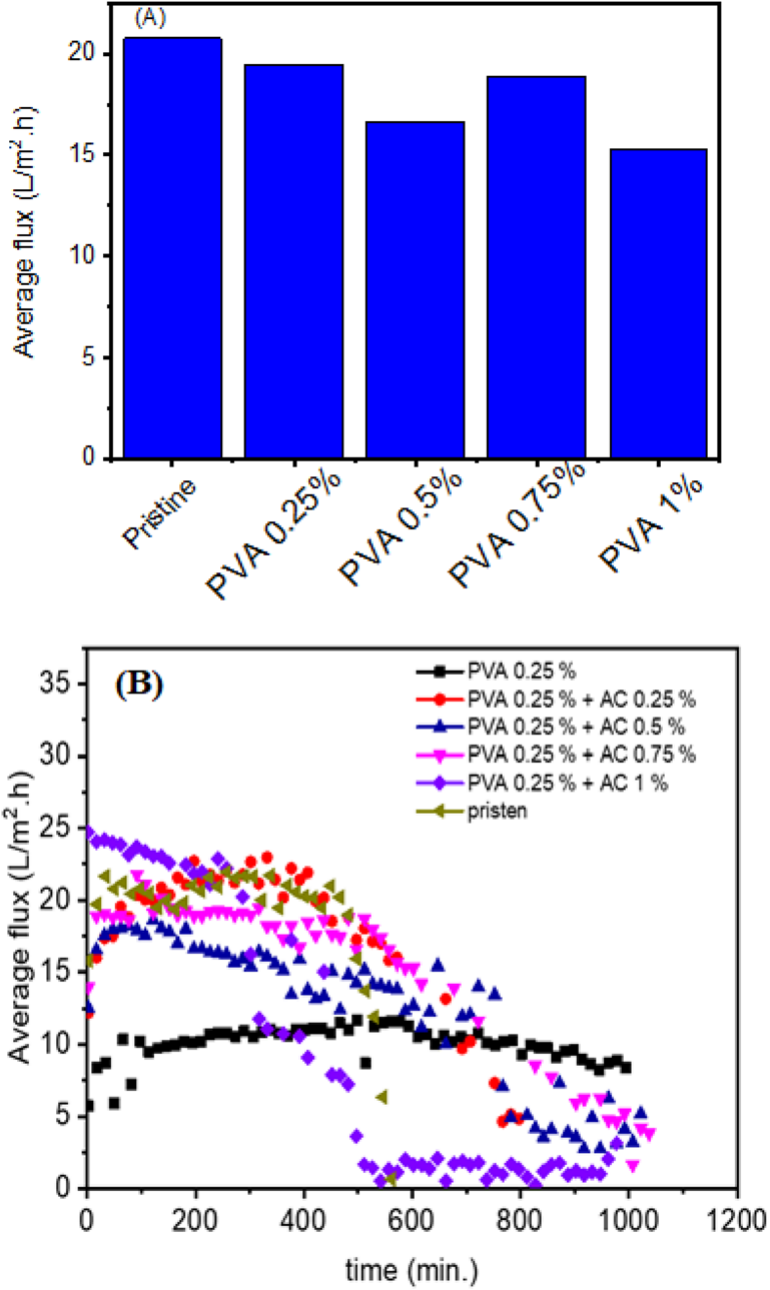
The graph illustrates the impact of varying base PVA concentrations on the average flux of membranes containing a constant AC 1% wt (A), the time-dependent flux behavior of PVA-based membranes with varying AC concentrations (B).

Factors like AC content and polymer concentration were studied using synthetic inland brine as a scale-inducing challenge to optimize coating conditions. The graph in Fig. 7A illustrates the impact of varying base PVA concentrations on the average flux of membranes containing a constant AC of 1 wt%. The pristine membrane exhibits the highest flux, indicating the inherent permeability of the unmodified membrane structure. Compared to the pristine membrane, flux is lower in all formulations when PVA and AC 1 wt% are added. This suggests that the presence of PVA, even with the addition of AC, reduces the overall permeability. Furthermore, the graph reveals a trend in flux as the base PVA concentration increases. The water flux tends to decrease as the base PVA concentration increases from 0.25% to 1% wt, despite the constant AC 1% wt content. This observation suggests that the higher the base PVA concentration, the denser or more compact the membrane structure becomes, leading to a greater resistance to vapor transport. It's noteworthy that even with the addition of AC 1% wt, which is expected to introduce porosity, the increasing PVA concentration still results in a decrease in flux. This implies that the effect of the increasing PVA concentration in reducing permeability outweighs the potential benefit of the added AC in enhancing it. This indicates that while AC can potentially enhance permeability, the base PVA concentration plays a critical role in determining the overall flux characteristics of the membrane. Also, the flux behavior of six different membrane formulations: a base membrane of 0.25% wt polyvinyl alcohol (PVA), this same PVA membrane with the addition of activated carbon (AC) at varying concentrations (0.25%, 0.5%, 0.75%, and 1% wt), and a pristine (unmodified) membrane. Fig. 7B clearly reveals the dynamic flux behavior of the membranes over time. The pristine membrane exhibits a relatively stable flux over the entire 1200-minute period. In contrast, the PVA 0.25% wt membrane shows a significantly lower and relatively stable flux compared to the pristine membrane. This indicates that even a low concentration of PVA coating reduces the membrane's permeability (from 0.53 to 0.2 kg m^−2^ h^−1^ °C^−1^), and the smoother surface morphology observed in SEM images indicate the formation of a denser layer that restricts solvent transport across the membrane.

The addition of activated carbon (AC) into the PVA matrix leads to a notable change in flux profiles. All AC-modified membranes initially show a higher flux compared to the 0.25% wt PVA membrane, suggesting that AC enhances permeability. The membranes with 0.25% wt and 0.5% wt AC show an initial rise in flux, followed by a gradual decline. This suggests that while AC initially facilitates vapor transport, potentially by creating pores, pore blockage may occur over time, leading to a decrease in flux. The membranes with 0.75% wt and 1% wt AC exhibit a more pronounced initial flux enhancement but also experience a more rapid and significant decline. This indicates that higher AC concentrations may initially offer more pathways for vapor transport but are also more susceptible to fouling or blockage.

Although the pristine PTFE membrane displayed a relatively higher flux, it lacks photothermal capability and tends to suffer from pore wetting during extended operation. The incorporation of activated carbon (AC) into the PVA matrix introduces photothermal functionality that enables efficient solar-to-thermal conversion, enhancing the thermal efficiency of the DCMD process. Moreover, AC contributes to improving the surface stability and anti-wetting properties of the coated membranes, which are crucial for long-term desalination performance. Therefore, the novelty of this work lies in developing a photothermally active and wetting-resistant PVA–AC composite membrane rather than focusing solely on maximizing the initial flux.

### 3.4. Photothermal MD operation

**Fig. 8:**
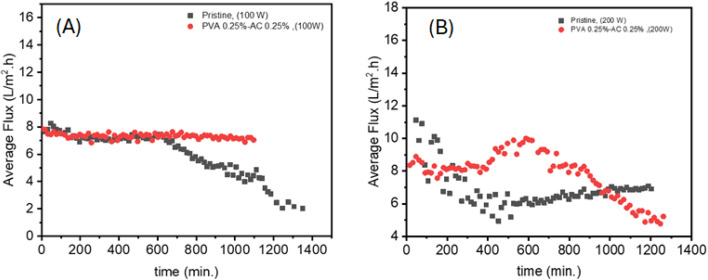
Time-dependent water vapor flux under simulated solar irradiation for pristine PTFE membrane, and PVA–AC (0.25–0.25%) wt coated membrane at 100 W m^−2^ (A), and at 200 W m^−2^ (B).

As mentioned before, coating membranes with AC is mainly driven by solar energy harvesting, which heats the membrane-feed interface locally to lower energy consumption in MD desalination.^50^ The performance of PVA–AC-coated membranes was evaluated, so MD was performed using light as the driving force, with a 30 °C temperature difference on the feed water. The membrane with PVA–AC coating showed better performance with higher light intensity. The performance of the pristine and PVA 0.25% wt–AC 0.25% wt coated membrane under different simulated light intensities is shown in Fig. 8. The pristine membranes exhibit a decline in flux over time, with a more pronounced decline at 200 W. The PVA–AC membranes show varying flux profiles, with a stable flux at 100 W and a more complex profile at 200 W. This suggests that processing power significantly influences membrane performance and stability.

The flux decline observed for the pristine PTFE at 200 W is likely due to uneven surface heating and partial pore wetting, as the PTFE lacks photothermal uniformity. The resulting thermal stress and condensation instability reduce the effective vapor transport compared to the more thermally balanced PVA–AC composite membranes.

### 3.5. Evaluation of the performance of the MD membranes

**Fig. 9:**
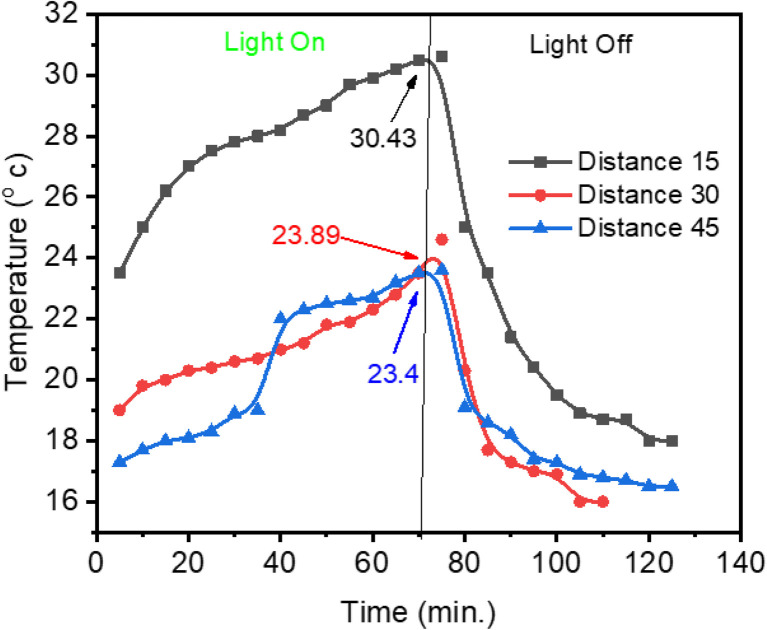
Effect of the light source distance on membrane surface temperature.

Light intensity is inversely proportional to the square of the distance from the source to the object; this is the inverse square law of light.^51^ Therefore, this part studied the effect of distance on the surface temperature of PVA–AC composite membranes used as photothermal material,^51^ which enables light-to-heat conversion. A tungsten lamp emitting light at 30 W m^−2^ served as the light source in this study. After illumination, the lamp was switched off, and the subsequent decline in temperature and flux was monitored to evaluate the thermal response of the membrane, as shown in Fig. 9. The light source distance, and its intensity, greatly affects the membrane's surface temperature, based on the results. As an example, a 15 cm distance from the light source led to a membrane temperature of 30.34 °C. On the other hand, the temperature was 23.89 °C at 30 cm and 23.4 °C at 45 cm.

The light ON/OFF test was carried out using the optimized PVA–AC–GA (0.25 wt% + 1 wt% + 1 wt%) membrane, which showed the most stable and reproducible photothermal response among all samples.

## 4 Conclusion

This study created a PVA–AC active layer on hydrophobic PTFE membranes. This membrane showed good salt resistance and sunlight-driven desalination. The coating achieved a high concentration factor without a large water vapor flux drop. This is promising for off-grid desalination systems handling fluids with high scaling potential. These advantages are important for brine concentration in inland areas. Minimizing waste brine production often requires significant water recovery. The membrane was used in thermal water desalination. The best flux membrane was selected. The developed PVA–AC–GA composite membrane demonstrated a permeability coefficient of 0.51 kg m^−2^ h^−1^ °C^−1^, close to that of pristine PTFE (0.58 kg m^−2^ h^−1^ °C^−1^), confirming its potential as a stable and photothermally active candidate for solar-assisted DCMD desalination. The study examined factors influencing membrane distillation. This included tests with and without light, including sunlight. The results confirmed the photothermal activity of the PTFE/PVA/AC membrane in photothermal MD. This is a promising improvement over conventional MD membrane.

## Conflicts of interest

The authors declare that they have no known competing financial interests or personal relationships that could have appeared to influence the work reported in this paper.

## Supplementary Material

RA-015-D5RA06460K-s001

## Data Availability

The data will be available on request. The data supporting this article have been included as part of the supplementary information (SI). Supplementary information is available. See DOI: https://doi.org/10.1039/d5ra06460k.
