# Supplementary material for: Development of a PTFE membrane with photo-thermal activated carbon nanomaterials for improved solar-driven membrane distillation
Source: RSC Adv. 2025 Oct 31;15(49):41946–58. doi: 10.1039/d5ra06460k (PMC12576948; doi:10.1039/d5ra06460k)
Supplement: RA-015-D5RA06460K-s001 [file RA-015-D5RA06460K-s001.pdf]

Supplementary Information (SI) for RSC Advances.
This journal is © The Royal Society of Chemistry 2025

Development of a PTFE Membrane with Photo-Thermal Activated Carbon Nanomaterials for Improved Solar-Driven Membrane Distillation

Wesam Abd El-Fattah[1], Ahlem Guesmi[1], Naoufel Ben Hamadi[1], Bassant Ebraheem[2], An Ding[3], Mohamed E.A. Ali[2,*]

[1]Chemistry Department, College of Science, Imam Mohammad Ibn Saud Islamic University (IMSIU), P.O. Box 5701, Riyadh 11432, Saudi Arabia.

[2]Egypt Desalination Research Center of Excellence (EDRC) & Hydrogeochemistry Dept., Desert Research Center, Cairo, 11753, Egypt.

[3]School of Environment and Energy Engineering, Beijing University of Civil Engineering and Architecture, Beijing 100044, China

Table (1): Represent Salts from a saline synthetic Solution.

Salt	Weight(gm)
**NaCl**	**24**
**Na_2So_4**	**4**
**$NaHSo_4$**	**0.172**
**$MgCl_2.6H_2O$**	**7.1**
**KCl**	**0.7**
**$CaCl_2$**	**1.14**

Table (2): percentages of the constituent elements of AC

Weight %

	C	N	O	Al	Si	Cl	Ca	Fe
ACTIVATED CARBON_pt1	28.9	2.8	40.6	11.6	13.9	0.2	0.4	1.7
ACTIVATED CARBON_pt2	36.4	7.3	39.6	4.0	12.2			0.5
ACTIVATED CARBON_pt3	43.3	15.8	32.3	2.8	4.3			1.6

Atom %

	C	N	O	Al	Si	Cl	Ca	Fe
ACTIVATED CARBON_pt1	39.3	3.2	41.6	7.0	8.1	0.1	0.2	0.5
ACTIVATED CARBON_pt2	45.8	7.9	37.4	2.2	6.5			0.1
ACTIVATED CARBON_pt3	51.3	16.0	28.7	1.5	2.2			0.4

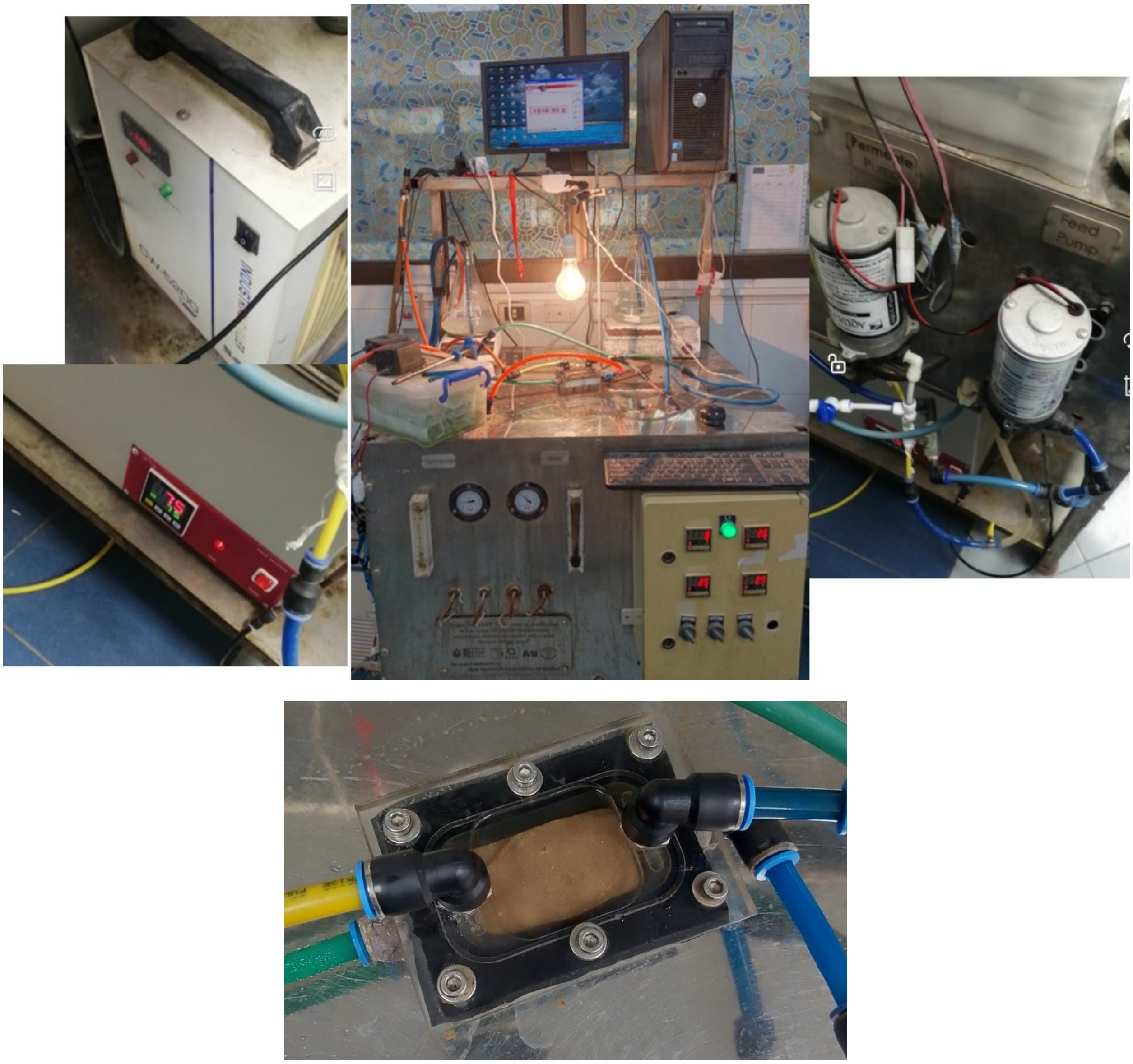

Fig.S1 Real form of the device ,and the used Cell with quartz window.
